# Study on Soy-Based Adhesives Enhanced by Phenol Formaldehyde Cross-Linker

**DOI:** 10.3390/polym11020365

**Published:** 2019-02-19

**Authors:** Zhigang Wu, Xuedong Xi, Hong Lei, Jiankun Liang, Jingjing Liao, Guanben Du

**Affiliations:** 1 Yunnan Provincial Key Laboratory of Wood Adhesives and Glued Products, Southwest Forestry University, Kunming 650224, China; wzhigang9@163.com (Z.W.); xuedongjx@163.com (X.X.); dushimensheng@126.com (J.L.); Jingjing_liao@hotmail.com (J.L.); 2 College of Forestry, Guizhou University, Guiyang 550025, China

**Keywords:** phenol formaldehyde, soy protein-based adhesives, cross-link, amino acid

## Abstract

To find the effects of cross-linker phenol-formaldehyde (PF) resin on the performance of soy-based adhesives, the reaction between model compounds hydroxymethyl phenol (HPF) and glutamic acid were studied in this paper. HPF prepared in laboratory conditions showed higher content of hydroxymethyl groups than normal PF resin, which was proved by the results of Electrospray Ionization Mass Spectrometry (ESI-MS) and ^13^C Nuclear Magnetic Resonance (^13^C-NMR). The results of ESI-MS, Fourier transform infrared spectroscopy (FT-IR), and ^13^C-NMR based on resultant products obtained from model compounds showed better water resistance of the soy protein-based adhesive modified by PF-based resin, which indicated the reaction between PF resin and soy protein. However, it seemed that the soy-based adhesive cross-linked by HPF with the maximum content of hydroxymethyl groups did not show the best water resistance.

## 1. Introduction

To meet with the needs of environmentally-friendly adhesives in the wood industry, more and more attention has been given to adhesives prepared with natural materials, such as soy protein-based adhesives. It is well-known that soy-based adhesives with no modification cannot be used directly for wood panels because of their poor water resistance [1,2]. Cross-linking modification has been proved to be one of the effective modification methods to resolve this problem. The cross-linkers include amino resins [3,4], a resultant derived from epichlorohydrin and ammonium hydroxide [5], polyethyleneglycol diacrylate [6], aldehyde and its derivatives [7], and others. Phenol-formaldehyde (PF) resin is widely used as a wood adhesive for the preparation of exterior wood panels for its good water resistance. It was also reported to be used to cross-link soy-based adhesives [8]. Yang developed a soy protein-based adhesive modified by PF with a solid weight ratio of PF/soy = 30:70 for plywood [9]. Another similar soy-based adhesive for Oriented Strand Board (OSB) was introduced by Hse [10]. Kreibich used a soy adhesive and a phenol-resorcinol-formaldehyde (PRF) adhesive to prepare finger-jointed boards with a “honeymoon” process [11]. The reports mentioned above proved that PF was an effective cross-linker of soy-based adhesives. However, almost all of these reports were mainly on the performances of the resulted panels and no report has been given on the reaction between PF resin and soy protein-based adhesive.

For soy-based adhesives prepared with the most commonly-used material, defatted soy flour, the idea that the soy protein in soy flour is the foundation for the preparation of the adhesive has been widely accepted. The reactive groups in soy-based adhesives normally include –OH, –SH, –COOH, and –NH_2_ from the amino acid units of soy protein. The modification of soy-based adhesives, especially the improvement on its water resistance, is presumed to be based on the reaction between one or some of these reactive groups from soy protein and cross-linkers. Although the reactive group might come from –NH_2_ of soy protein according to the chemical structure of PF resin, there has been no direct proof or report.

The analysis on the reaction between protein and PF cross-linker seems impossible using modern instruments with the samples prepared from soy flour, which is a complicated mixture with some ingredients. Even for soy-based adhesive prepared with soy protein isolate (SPI), whose protein content is higher than 90%, the analysis on chemical structures of resulted adhesive is also very difficult because of the complicated amino acid compositions of soy protein. Although FT-IR was always used to analyze the chemical structures of soy-based adhesives [12], the information it could give was rather limited. All things considered, an amino acid, specifically glutamic acid, was chosen as a model compound to study the modification mechanism of soy adhesives by PF resin. According to the result of Tilley on the amino acid compositions in soy protein [13], glutamic acid covers 23.75 mol % in β-conglycinin, the most important composition of SPI, and stands out of the 16 measured amino acids. The most important point is that the application of pure glutamic acid makes the analysis on the reaction between protein and cross-linker possible. Unlike soy protein with some reactive groups, the reactive group in PF resin comes mainly from hydroxymethyl. Therefore, the hydroxymethyl phenol (HPF) with higher content of hydroxymethyl groups than a normal PF resin was used as a model compound of PF resin in this work.

Although PF resin is toxic, it was still used as a cross-linker of soy-based adhesives in this work for three reasons: (1) PF was proved to be useful for the cross-linking of soy-based adhesives. (2) As the first synthetic resin in industry, the chemical structure and performances of PF are relatively simple. (3) As an important aldehyde derivative, a study on the interaction between PF and soy protein will shed some light on the modification of soy-based adhesives by aldehyde and its derivatives.

The novelty of this work is mainly due to the usage of the model compound glutamic acid. Our work on the reaction between HPF and a common dipeptide has already been reported [8]. The reaction between the unit composition of the protein’s amino acid and hydroxymethyl phenol will be more helpful for the understanding of the reaction between soy protein and hydroxymethyl phenol.

## 2. Materials and Methods

### 2.1. Materials

The soy flour with 53.4% protein content was from Yuxin Soybean Protein Co., Ltd., Shandong, China. The thickness of poplar veneer for plywood was 1.5 mm and its moisture content was 8–10%. The formaldehyde solution with a concentration of 37 wt % was from Sinopharm Chemical Reagent Co., Ltd., Shanghai, China, and the formaldehyde solution of 50 wt % was from Kunming Xinfeilin Panel Board Co., Ltd., Kunming, China. l-glutamic acid was purchased from Shanghai Kangda Amino Acid Factory (Shanghai, China), with a purity of 98.5%.

### 2.2. Preparation of PF Resin-Based Cross-Linker

The PF resin with molar ratio F/P = 3.0 was prepared as follows: 94 parts by mass phenol, 28 parts deionized water, and 243 parts by mass formaldehyde 37% was charged into a flat bottom flask equipped with a condenser, thermometer, and a magnetic stirrer bar. The temperature was then slowly increased to 94 °C over a period of 30 min. The pH was adjusted to 8.5–9.0 with NaOH 30%. After being kept at 94 °C for 60 min, the mixture was then cooled naturally to room temperature to yield a yellow transparent resin with viscosity 750 cps and solid content 45.2%.

To retain more hydroxymethyl reactive groups, hydroxymethyl phenol (HPF) was prepared with the same molar ratio as the PF resin above to be used to study the effects of the content of hydroxymethyl on the performance of soy protein-based adhesive. HPF was prepared as the reference [8]: 94 parts by mass phenol and 180 parts by mass formaldehyde 50% was charged into a flat bottom flask equipped with a condenser, thermometer, and a magnetic stirrer bar. The pH was adjusted to 8.5–9.0 with NaOH 30%. After stirring at 30 ± 1 °C for 12, 24, and 48 h, the samples with the name of HPF-a, HPF-b, and HPF-c, respectively, with 43.0%, 44.6%, and 45.1% solid content were obtained.

### 2.3. Preparation of Soy-Based Adhesive

A soy-based adhesive was prepared according to [14]: 320 parts water and 80 parts by mass soy flour was put into a three-neck round-bottom flask equipped with a mechanical stirrer, thermometer, and condenser. Under continuous stirring, the mixture was heated to 45 °C. Then, 21.3 parts NaOH 30% was added to the flask. After 30 min, 20 parts urea solution 40% was added. After another 20 min, the soy protein-based adhesive with the name DS was obtained. Its solid content was 24 ± 1%.

### 2.4. Verification Experiment on Reaction between PF Resin and Soy-Based Adhesive

Samples of DS, DS with PF resin (PF/DS), and DS with HPF-a, HPF-b, HPF-c (HPF-a/DS, HPF-b/DS, HPF-c/DS) were dried in an oven at 180 °C to a constant weight. After being ground into a fine powder, m_1_ g of each sample was immersed in boiling water for 40 min. The residue was filtered, dried, and weighed as m_2_ g. The water resistance of the samples was characterized as ω%, which was defined as the ratio of m_2_/m_1_.

### 2.5. Preparation of Plywood with Soy Protein-Based Adhesive

The soy protein-based adhesive was mixed well with cross-linkers just before the preparation of three-layer plywood with a dimension of 300 mm × 220 mm × 4 mm. The solid addition amount of PF resin cross-linkers was 12% of the solid soy adhesive. During the preparation of the plywood, the veneers with double side adhesive loading of 320 g/m^2^ were rested at room temperature for 15 min. The assembled veneers were then sent into a XLB type single-layer hot press from Shanghai Rubber Machinery Plant (Shanghai, China) and pressed under a pressure of 1.5 MPa at 180 °C for 5 min to get a plywood sample.

### 2.6. Test of Dry and Wet Shear Strength of Plywood Samples

Before being cut into shear specimens with dimensions of 100mm × 25mm, the plywood panel was conditioned at (20 ± 2) °C and with relative humidity (65 ± 5)% in the laboratory for 1 day. The bonded area of each specimen was 25 mm × 25 mm. A WDS-50KN mechanical testing machine (Qingdao, China) was used to test both dry and wet shear strength of the plywood specimens. The testing method for dry and wet shear strength was performed with reference to Chinese national standard GB/T 9846.3-2004 and GB/T 17657-1999, respectively. According to the standard, the final wet shear strength was 0.9 times of the testing strength of the specimens after being immersed into boiling water for 3 h. The dry or wet shear strength was from the mean result of 8–10 specimens.

### 2.7. ESI-MS

PF resin-based cross-linkers, GA, and the modified soy based adhesive were respectively dissolved in chloroform to get a solution with a concentration of 10 μL/mL, that was 10 μL sample and 1 mL chloroform. The solution was then injected into the ESI source plus ion trap mass spectrometer via a syringe at a flow rate of 5 μg/s. Spectra were recorded in positive mode with ion energy of 0.3 eV and scan range of 0–1000 Da on a Waters Xevo TQ-S instrument (Milford, MA, USA).

### 2.8. ^13^C-NMR

A 400 μL sample was dissolved in 200 μL dimethyl sulfoxide-d6 (DMSO-d6). A liquid ^13^C-NMR (zgig) spectrum was then obtained from a Bruker Avance spectrometer with a frequency of 600 MHz (Billerica, MA, USA). The chemical shifts were calculated relative to the control (CH_3_)_3_Si(CH_2_)_3_SO_3_Na dissolved in DMSO-d_6_. The spectra were performed at 39062.5 Hz for a number of transients of 500–800. All the tests were run with a relaxation delay of 6 s and the chemical shifts were accurate to 1 ppm. NMR spectra were integrated. The functional groups were quantified and calculated as follows [15,16,17]: With methylene glycol bond at 83 ppm as benchmark peak, integral on all absorption peak. Then, to sum all of the methylene carbon integral areas (besides methanol peak at 50 ppm and methoxyl ether peak at 56 ppm), the percentage of integral values of various types of chemical bonds to total methylene carbon were calculated.

### 2.9. FT-IR

Some of the liquid soy-based adhesives with or without cross-linkers were put into an oven with a temperature of 160 °C. After curing to a constant weight, the soy-based adhesive samples were ground into fine powder, and 0.001 g of each sample was mixed well with 1 g KBr to prepare a pill. Then, the dried pill was tested in a Varian 1000 infrared spectrophotometer (Palo Alto, CA, USA).

## 3. Results and Discussion

### 3.1. The Chemical Structure of Hydroxymethyl Phenol

**Figure 1:**
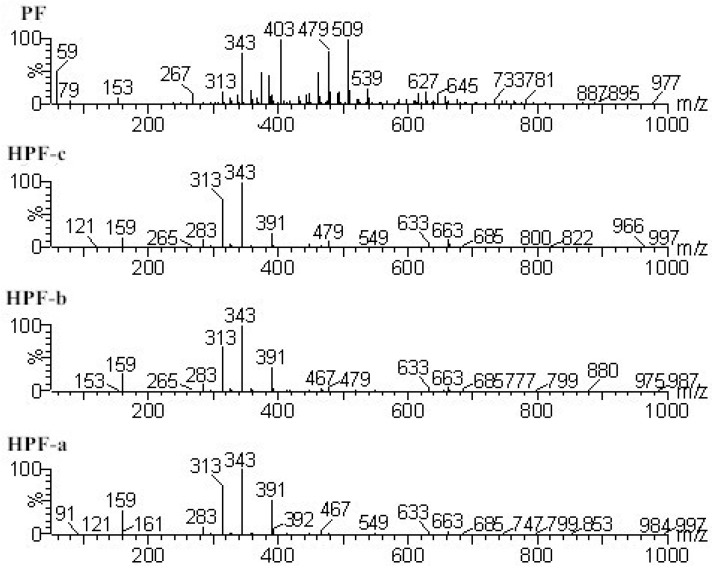
Electrospray Ionization Mass Spectrometry spectra of samples HPF-a, HPF-b, HPF-c and phenol-formaledhyde.

The hydroxymethyl in PF resin was thought to be the main reactive group and finally affect the properties of soy protein-based adhesives. Both HPF with higher content of hydroxymethyl groups and a normal PF resin were prepared in this work. The ESI-MS spectra of the three HPF samples and the normal PF resin were shown in Figure 1. The main ion peaks obtained from ESI-MS spectra and their assignment can be seen from Table 1.

The ESI-MS spectra of all the three HPF samples, namely HPF-a, HPF-b, and HPF-c, looked very similar. The ion peaks mainly came from 283, 313, 343, and 391 Da, which were all assigned as dimers with two phenol molecules, while for normal PF resin, the ion peaks mainly occurred at 343, 391, 403, 479, and 509 Da, which meant that both dimmers were shown, as in HPF samples, and even some trimers or tetramers in the PF resin sample. In all, the ESI-MS results proved the lower polymerization degree of HPF samples than that of the PF resin sample, which might be good for the cross-linking of soy protein.

**Figure 2:**
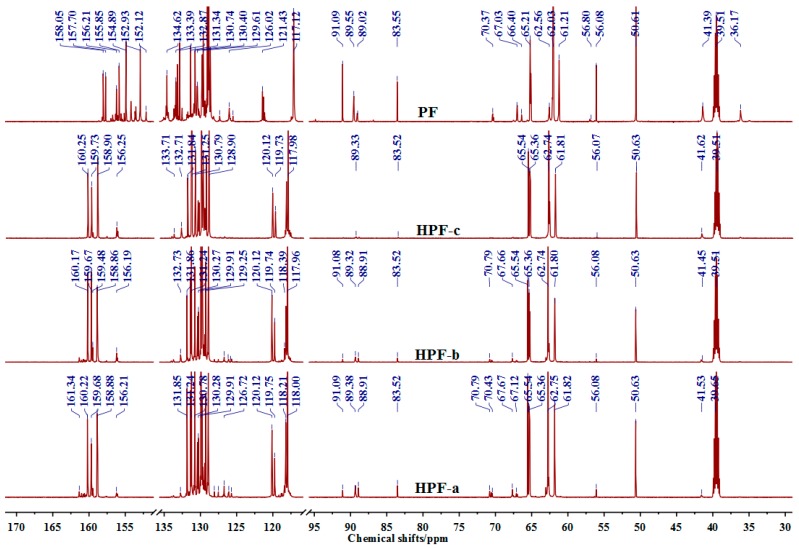
The ^13^C-NMR spectra of samples PF, HPF-a, HPF-b, and HPF-c.

The ^13^C-NMR spectra of HPF and PF samples are given in Figure 2 and their quantitative results are in Table 2. The four ^13^C-NMR spectra looked very similar, which were easy to be understood when considering the same materials for the sample preparation and their similar chemical reaction. The main difference of these samples came from the content of hydroxymethyl groups, which were 85.1%, 89.9%, 94.9%, and 55.1% for HPF-a, HPF-b, HPF-c, and PF samples, respectively, calculated by methods mentioned in this paper. It indicated that the content of hydroxymethyl groups of HPF was all higher than that of the normal PF resin. The longer the reaction time, the more hydroxymethyl groups would be obtained. Both the ^13^C-NMR and the ESI-MS results showed the high content of hydroxymethyl groups in HPF samples prepared in this work.

### 3.2. The Interaction between HPF and Soy Protein

A verification experiment was done to prove the interaction between PF resin and soy protein-based adhesive. The results are given in Table 3. According to the design of the experiment, the ω was obtained as the total weight divided by the insoluble parts of soy adhesives. It represented the water resistance of the soy-based adhesives. As seen in Table 3, the water resistance of soy-based adhesive improved greatly after mixing with HPF and PF. It was obvious that the soy-based adhesive without cross-linker had no water resistance. The residue of soy-based adhesives with cross-linker (m_2_) was more than the pure HPF or PF (solid weight of cross-linker), which was a good indication of the interaction between soy-based adhesive and HPF or PF. One thing has to be pointed out—if the content of soy protein was at a normal level (50% in the soy flour used in this work), the weight of the residue from sample HPF-b/DS was even higher than the total solid amount of soy protein and cross-linker. It seemed that HPF might react with soy proteins and even non-proteins in a soy-based adhesive, such as a carbohydrate. The soy protein-based adhesive cross-linked with HPF-b containing medium content of hydroxymethyl groups showed more insoluble residue rather than HPF-c with more hydroxymethyl groups. It might be related to the complicated components of soy-based adhesive, especially after the soy flour’s hydrolysis.

**Figure 3:**
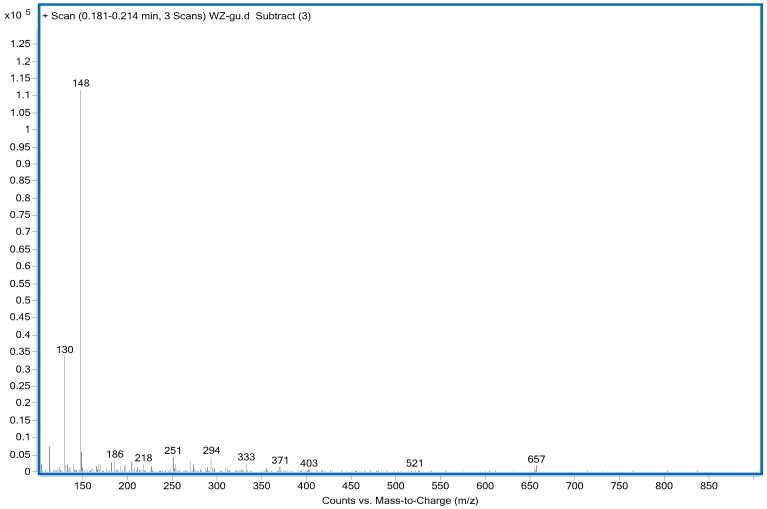
ESI-MS spectra of sample l-glutamic acid.

**Figure 4:**
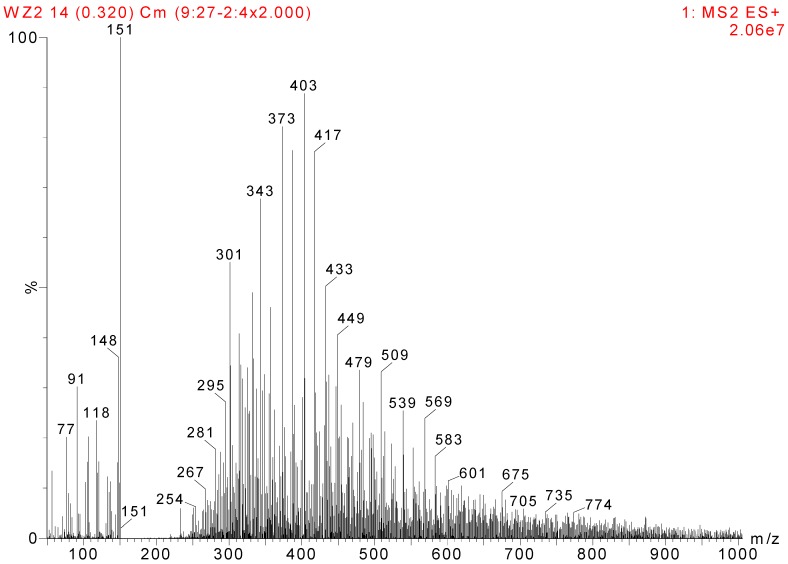
ESI-MS spectra of sample hydroxymethyl phenol/l-glutamic acid.

Glutamic acid (GA) was chosen as a model compound to study the interaction between HPF and soy protein. The ESI-MS spectra of GA and HPF/GA are given as Figure 3 and Figure 4, respectively. The assignments of their main ESI-MS ion peaks can be seen in Table 4. For a chemical group with a nitrogen atom, the ion peak of ESI-MS will normally be observed with a form of M + H^+^. By calculation, the peak at 148 Da came from the GA molecules. The peak at 295 Da was the addition product from one molecule of GA and one molecule of hydroxymethyl phenol. The peak at 417 Da was assigned as a complex with Na^+^ composed of one molecule of GA and two molecules of hydroxymethyl phenol. The peaks of 449, 479, 509, 539, and 569 Da with an interval of 30 Da were from the addition reaction of one molecule of GA and a dimer of HPF resin with two molecules of phenol. The difference between them was mainly in the quantities of hydroxymethyl groups. Table 4 proved the reaction between GA and HPF.

**Figure 5:**
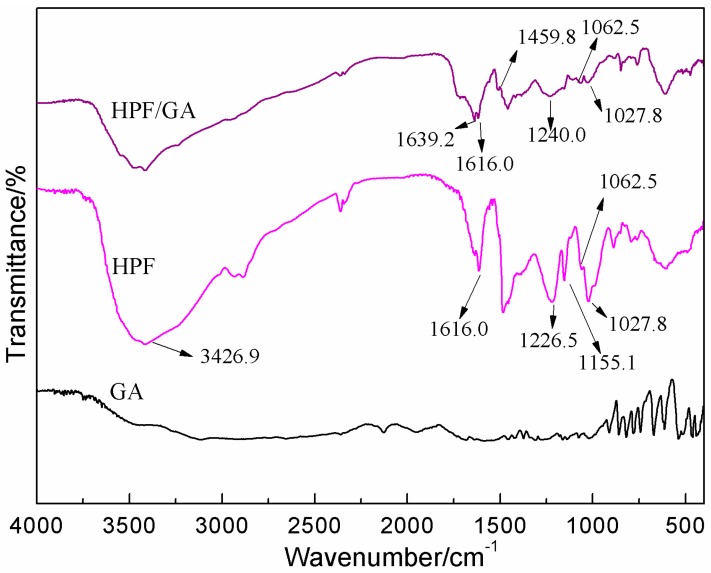
FT-IR spectra of GA, HPF-b, and HPF-b/GA.

The FT-IR spectra of samples GA, HPF-b, and their mixture (HPF-b/GA) are given as Figure 5. For HPF, the broad band observed in 3462.9 cm^−1^ was assigned to the bound O–H groups. The absorption band of C=C from the phenyl ring was observed at 1616.0 cm^−1^. The absorption of C–C stretching from the bonding of the phenolic ring and hydroxymethyl group was seen at 1155.1 cm^−1^. The absorption of C–O of phenolic hydroxyl groups, ph–C–O groups, and hydroxymethyl groups was seen at 1226.5, 1062.5, and 1027.8 cm^−1^, respectively [18,19].

In the FT-IR spectrum of HPF-b/GA, the absorption of C–O of phenolic hydroxyl groups moved from 1226.5 to 1240.0 cm^−1^. The intensified cloud density on the phenolic ring caused by the reaction between hydroxymethyl and soy protein might be responsible for the shift [20].

A new absorption at 1459.8 cm^−1^ appeared in the FT-IR result of the sample HPF-b/GA, which could be assigned to the C–N stretching vibration obtained from the reaction between hydroxymethyl groups from HPF and amino groups from soy protein [21].

After mixing with GA, the absorption at 1027.8 cm^−1^, which was assigned to the C–O from hydroxymethyl groups, decreased. It meant that large quantities of the hydroxymethyl groups were consumed after mixing with GA. At the same time, the absorption from ph–C–O groups at 1062.5 cm^−1^ increased. Since the reaction among hydroxymethyl phenol was small or even invisible for its high activation energy [22,23,24], the observed increase of the absorption of ph–C–O should be caused by the new C–O groups from the reaction between hydroxymethyl phenol and GA. For GA, the reactive groups might come from both –COOH and –OH groups.

**Figure 6:**
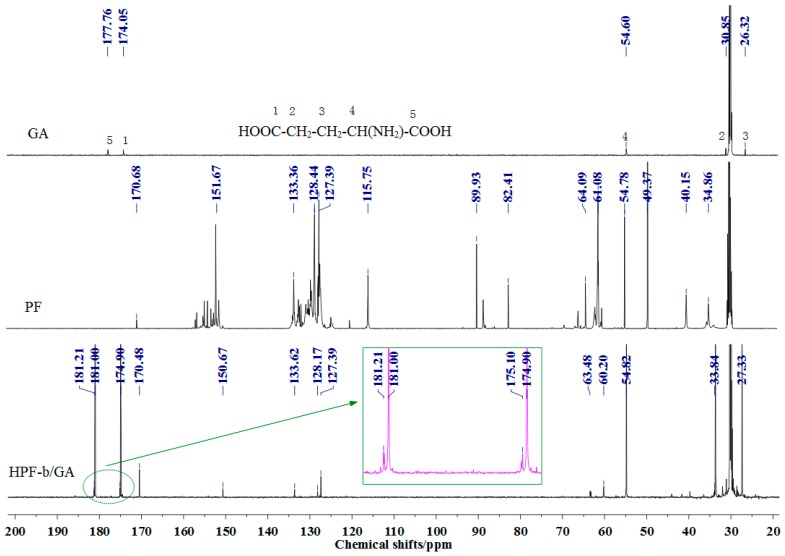
The ^13^C-NMR spectra of sample GA, PF, and HPF-b/GA.

The ^13^C-NMR spectra of samples GA, HPF-b (PF), and HPF-b/GA are showed in Figure 6. A magnified figure in the range of 172–182 ppm of sample HPF-b/GA was also given. The labelled five absorptions in the ^13^C-NMR spectra of the GA sample were assigned as five carbons of GA with different chemical environments, respectively, seen from the GA’s chemical formula given in Figure 6. The absorptions from two carboxyl groups were seen exactly at 174.05 and 177.76 ppm. However, for the sample HPF-b/GA, several more peaks were seen around 174 and 177 ppm, which indicated the change of the charge density of carbonyl groups because of the reaction on carboxyl groups.

**Scheme 1:**
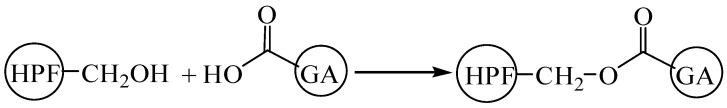
The possible reaction between phenol-formaldehyde or hydroxymethyl phenol and l-glutamic acid.

According to the analysis on the results of ESI-MS, FT-IR, and NMR, the possible reaction between PF or HPF and GA was shown in Scheme 1.

It also represents the possible reaction when using PF or HPF to cross-link soy protein-based adhesives.

### 3.3. Performance of Soy Protein-Based Adhesives Cross-Linked by Hydroxymethyl Phenol

The performance of plywood prepared with soy-based adhesive cross-linked by HPF and PF is shown in Table 5. The dry shear strength for all of the plywood specimens with soy-based adhesives was good enough to meet with the requirement of the relative Chinese national standard (GB/T 9846.3-2004, ≥0.70MPa). In fact, even for those without a cross-linker, there was no problem for the dry shear strength to satisfy the requirement. The cross-linkers were mainly added to improve the water resistance of soy protein-based adhesives. As seen in Table 5, the wet shear strength of the adhesives indeed increased with the addition of PF or HPF. The immeasurable wet shear strength indicated the poor water resistance of the soy-based adhesive without a cross-linker. However, all of the wet shear strengths of soy protein-based adhesive with PF or HPF satisfied the relative standard (GB/T 9846.3-2004, ≥0.70MPa). As seen in Table 5, the specimens with HPF-b showed the highest wet shear strength, which was consistent with the result of Table 3.

## 4. Conclusions

To find the effects of cross-linker phenol-formaldehyde (PF) resin on the performance of soy-based adhesives, the reaction between model compounds hydroxymethyl phenol (HPF) and glutamic acid were studied in this paper. HPF prepared in the laboratory condition showed higher content of hydroxymethyl groups than a normal PF resin, which was proved by the results of ESI-MS and ^13^C-NMR. The results of ESI-MS, FT-IR, and ^13^C-NMR based on resultant products obtained from model compounds showed better water resistance of the soy protein-based adhesive modified by PF-based resin, which indicated the reaction between PF resin and soy protein. However, it seemed that the soy-based adhesive cross-linked by HPF with the maximum content of hydroxymethyl groups did not show the best water resistance.

## Figures and Tables

**Table 1:** The main ion peaks of ESI-MS of PF resin-based cross-linker and their assignment.

M+Na^+^ (Experimental) Da	Calculated Units
159	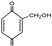
283	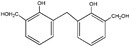
313	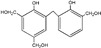
343	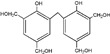
391	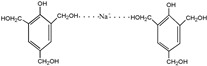
479	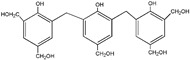
509	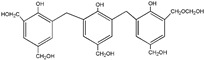
539	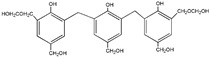
615	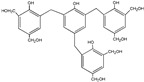
627	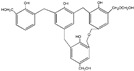
645	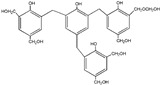

**Table 2:** Methylene carbons contents in different samples calculated from results of ^13^C-NMR.

Structure	Chemical Shift (ppm)	HPF-a	HPF-b	HPF-c	PF
*o*–CH_2_–p	34–36	0.0	0.0	0.8	18.0
p–CH_2_–p	39–41	1.2	1.6	3.2	10.4
*o*–CH_2_OH HOCH_2_–*o*–CH_2_OH Ph–(CH_2_OH)_3_–/–	61.4–65.52	85.1	89.9	94.9	55.1
Ph–(CH_2_O)_n_H(n ≥ 2)	69.1–70.07	3.6	2.3	0.0	4.9
Ph–CH_2_–O–CH_2_–Ph	72–74	2.0	1.2	0.0	1.1
HOCH_2_OH	83.73	1.3	0.8	0.0	1.7
formaldehyde and its derivatives	84–92.87	6.8	4.2	1.1	8.7

**Table 3:** Verification test of reaction between PF cross-linker and soy-based adhesive.

Samples	Solid Weight of Soy-Based Adhesive/g	Solid Weight of Cross-Linker/g	m_1_/g	m_2_/g	Ω (m_2_/m_1_)/%
DS	2.00	0.00	2.00	0.00	0
HPF-a/DS	1.79	0.21	2.00	1.14	57
HPF-b/DS	1.79	0.21	2.00	1.32	66
HPF-c/DS	1.79	0.21	2.00	1.10	55
PF/DS	1.79	0.21	2.00	1.00	50

**Table 4:** The main ion peaks of ESI-MS of GA and HPF/GA and their assignment.

Samples	M+H^+^ (exp.)	Calculated Units
GA	148	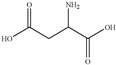
HPF/GA	295	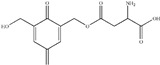
	417	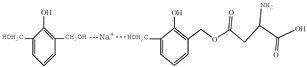
	449	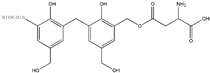 *n* = 1,2,3,4,5
	479	
	509	
	539	
	569	

**Table 5:** The bonding performance of soy protein-based adhesives.

Adhesive	Viscosity/mPa·s	Dry Shear Strength/MPa	Wet Shear Strength/MPa
DS	280	0.98(0.06)	-
HPF-a/DS	440	1.82(0.16)	0.98(0.06)
HPF-b/DS	396	1.81(0.12)	1.18(0.08)
HPF-c/DS	310	1.68(0.13)	0.82(0.06)
PF/DS	1020	2.04(0.15)	0.84(0.03)
